# Photocleavable Systems for Cell Biology: Conceptual Design across Molecular Modalities

**DOI:** 10.1002/cbic.202500564

**Published:** 2025-10-29

**Authors:** Masahiko Yoshimura, Tomoko Inose

**Affiliations:** ^1^ Institute for Integrated Cell‐Material Sciences (WPI‐iCeMS) Institute for Advanced Study Kyoto University Yoshida, Sakyo‐ku Kyoto 606–8501 Japan; ^2^ The Hakubi Center for Advanced Research Kyoto University Yoshida, Sakyo‐ku Kyoto 606–8501 Japan; ^3^ JST PRESTO Saitama 332–0012 Japan; ^4^ Department of Synthetic Chemistry and Biological Chemistry Graduate School of Engineering Kyoto University Katsura, Nishikyo‐ku Kyoto 606–8502 Japan

**Keywords:** chemical biology, coumarin, photochemistry, photocleavage, photolysis

## Abstract

The spatiotemporal control of biomolecular functions via light‐triggered bond cleavage has emerged as a powerful approach in chemical biology and cell biology. In this concept review, three major modalities of photo‐cleavable systems—proteins, small molecules, and metal complexes—are classified and discussed, highlighting their design principles, biological applicability, and remaining challenges. Emphasis is placed on recent efforts to address key design challenges—such as balancing functional performance, biological compatibility, and optical responsiveness—across different molecular modalities, offering perspectives for the next generation of photo‐responsive tools for biological research.

## Graphical abstract

Light‐triggered bond cleavage is an essential biotechnology in chemical biology research. This concept highlights three photocleavable modalities—proteins, small molecules, and metal complexes—distilling design principles for wavelength tuning, cleavage efficiency, and biocompatibility. By comparing strengths/limits, we discuss the next generation of photo‐responsive tools for biological research.

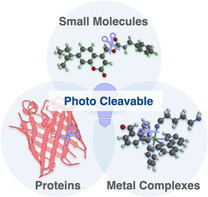

## 1 Introduction

**Figure 1:**
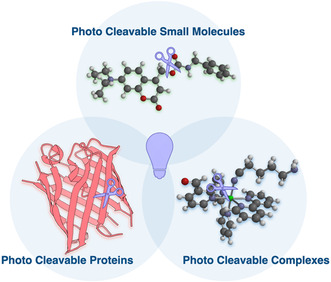
Photocleavable modalities can be broadly classified into three conceptual categories: photocleavable proteins, photocleavable small molecules, and photocleavable metal complexes. Each represents a distinct strategy for light‐triggered bond cleavage and biological control.

Photo‐responsive molecules are useful tools for spatiotemporal control of molecular events.^[^ ^1^, ^2^, ^3^, ^4^, ^5^, ^6^, ^7^, ^8^, ^9^, ^10^ ^–^ ^11^ ^]^ In particular, molecules capable of light‐triggered bond cleavage have emerged as powerful tools for the irreversible control of molecular functions. These technologies are now widely applied across diverse fields, from materials science to cellular biology.^[^ ^12^, ^13^, ^14^, ^15^, ^16^, ^17^ ^–^ ^18^ ^]^ Due to their ability to exert spatiotemporal control over molecular functions in complex cellular environments, photocleavable molecules have become indispensable tools in chemical and molecular biology. The origin of photocleavage technology dates back to 1962, with the discovery of photoinduced bond cleavage in benzyloxycarbonylglycine.^[^ ^19^ ^]^ While early systems exhibited limited photolytic efficiency and were primarily used in fundamental photochemical studies, subsequent advances in molecular design and photophysical characterization have significantly expanded their utility. Modern photocleavable systems now include a broad range of organic molecules, as well as metal complexes and genetically encoded protein‐based platforms. This review outlines the evolution of photocleavable modalities—including small molecules, coordination complexes, and proteins—highlighting their molecular design principles, functional advantages and limitations, and practical applicability in the context of cellular function manipulation (**Figure** **1**).

## 2 Photocleavable Proteins

Among various strategies for optical control in living cells, the use of light‐responsive proteins represents one of the most rational and accessible approaches. For biologists familiar with genetic manipulation, the development and use of functional proteins are relatively accessible, contributing to their widespread use. Moreover, their endogenous expression allows them to avoid delivery‐related challenges faced by external photoreactive molecules, including solubility, membrane permeability, and subcellular localization. This section reviews recent advances in protein‐based photocleavage platforms, focusing on two principal light‐responsive systems, photocleavable protein (PhoCl)^[^ ^20^ ^]^ and light‐assisted uncaging switch for endoproteolytic release (LAUNCHER).^[^ ^21^ ^]^

### 2.1 PhoCl

**Figure 2:**
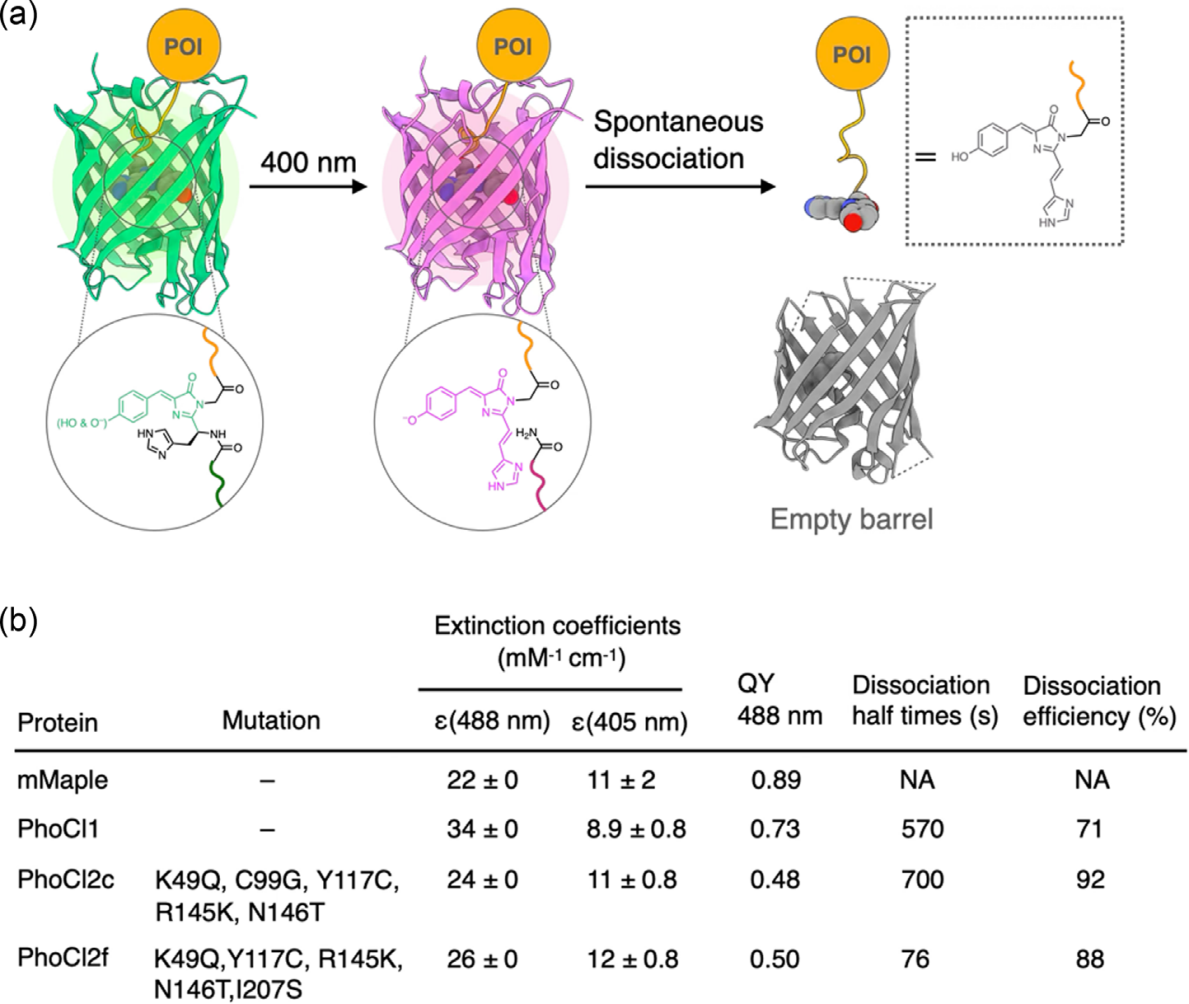
PhoCl system, protein‐based light‐triggered bond cleavage. a) A mechanism of light‐triggered bond cleavage in PhoCl. b) Spectral properties of PhoCl variants. QY means quantum yield.

PhoCl is a genetically encodable, photolytically cleavable protein that enables precise spatiotemporal regulation of intracellular proteins. Campbell and coworkers first recognized in 2017 that the green‐to‐red photoconvertible fluorescent protein mMaple undergoes a violet‐light‐induced *β*‐elimination that cleaves the polypeptide backbone adjacent to its chromophore.^[^ ^22^ ^]^ By circularly permuting mMaple, creating variant libraries, and applying directed evolution, they produced PhoCl—a construct that self‐dissociates upon ≈ 400 nm irradiation and releases a C‐terminal peptide fragment, with a fragment‐dissociation half‐time of ≈500 s (**Figure** **2**a). Although functionally useful, first‐generation PhoCl often suffered from inefficient photocleavage. Building on X‐ray crystal structures of the green, red, and cleaved “empty‐barrel” states, Campbell's group combined molecular‐dynamics insights with structure‐guided site‐saturation mutagenesis and a NanoLuc complementation screen to evolve second‐generation variants.^[^ ^23^ ^]^ PhoCl2f, for example, incorporates mutations in and near the 201–207 loop and dissociates roughly seven‐fold faster (fragment dissociation half‐time, t_1/2_ ≈ 76 s, measured after photoirradiation), while PhoCl2c provides higher photocleavage efficiency, with gel‐filtration analysis showing that ≈92% of the PhoCl2c fusion proteins undergo complete dissociation upon photoirradiation, compared to ≈71% for the first‐generation PhoCl and ≈88% for PhoCl2f (Figure 2b). The improved kinetics of PhoCl2 variants have broadened their utility. A 2024 study leveraged Golgi‐anchored PhoCl2c fusion payload proteins, using brief 405 nm pulses to uncage the payload proteins and thereby reconstituted ion‐channel function and downstream signaling pathways in living cells.^[^ ^24^ ^]^ Together, these advances establish PhoCl and its variants as versatile optogenetic tools for irreversible, light‐activated protein uncaging in diverse cellular systems.

### 2.2 Launcher

**Figure 3:**
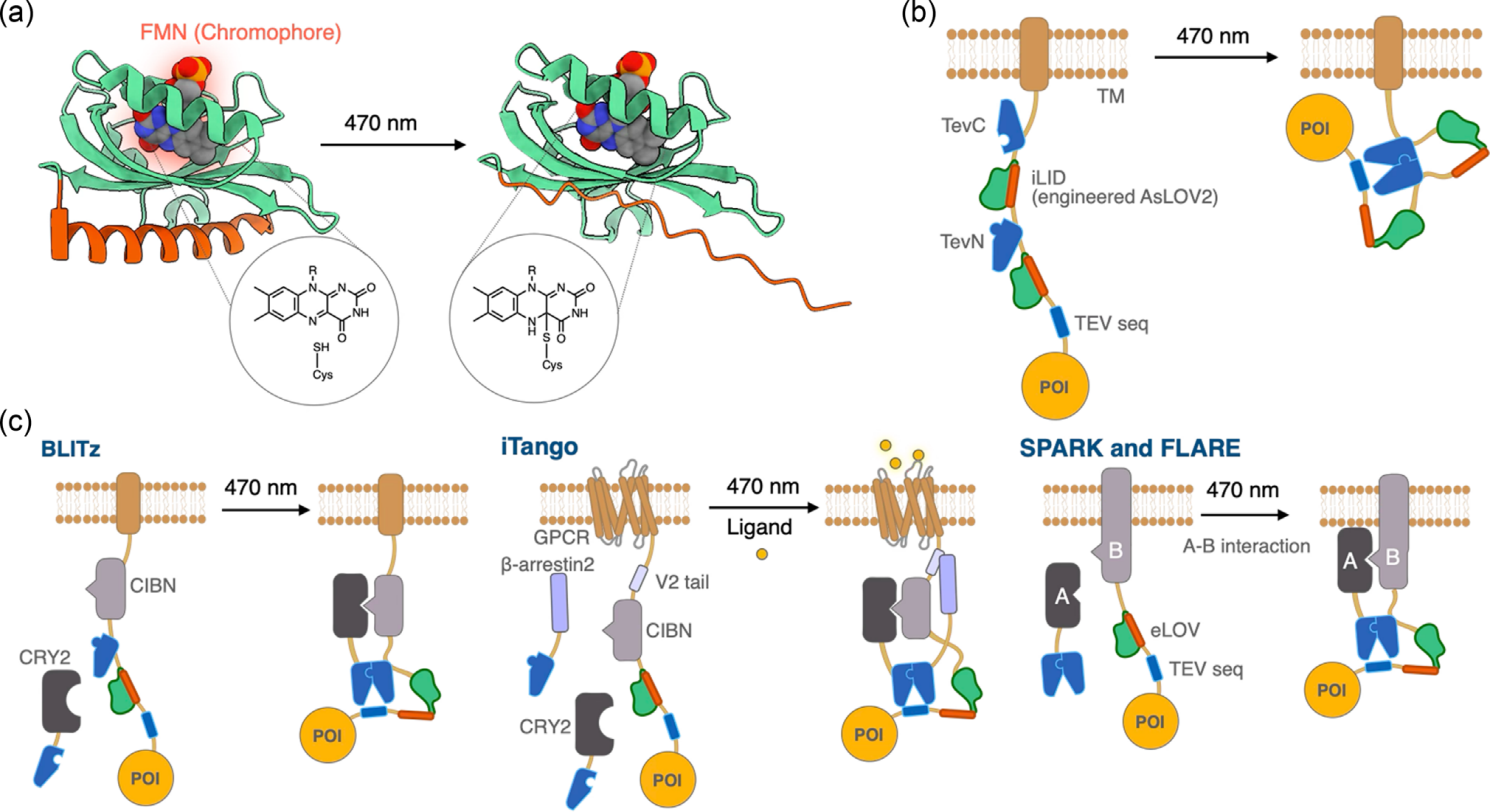
LOV2‐based light‐triggered bond cleavage systems. a) Photoreaction of FMN with a cysteine residue on LOV2 and conformational change of LOV2. b) Schematic illustration of LAUNCHER. c) Schematic illustrations of LOV2‐based photoresponsible systems, BLITz, iTango, and SPARK/FLARE.

Combining photo‐responsive proteins with proteolytic enzymes has yielded molecular systems in which light irradiation cleaves specific peptide bonds. The most widely used photosensor in this context is the second light‐oxygen‐voltage domain (LOV2) of the plant photoreceptor phototropin.^[^ ^25^, ^26^ ^–^ ^27^ ^]^ LOV2 holds a flavin mononucleotide (FMN) cofactor; blue‐light (≈470 nm) illumination induces a covalent adduct between FMN and a cysteine residue on LOV2, triggering a drastic conformational change (**Figure** **3**a). In 2024, Lee et al. harnessed a LOV2 variant—iLID (improved Light‐Induced Dimer)—to create LAUNCHER, a photocleavage system built by tandemly inserting iLID into a split TEV protease (TEVC–iLID–TEVN–iLID) followed by a TEV recognition site and a payload protein (Figure 3b).^[^ ^21^ ^]^ Light‐driven structural rearrangement of the two iLID modules reconstitutes the split protease and facilitates access to the TEV recognition site, which then cleaves the recognition sequence and releases the payload. LOV2‐based photo‐responsive architectures now exist in many formats, including hybrid designs that couple light with small‐molecule binding or intermolecular interactions; prominent examples such as BLITz, iTango,^[^ ^20^ ^]^ and SPARK/FLARE^[^ ^28^ ^,^ ^29^ ^]^ have been applied to control protein localization (Figure 3c).

This section has highlighted two cutting‐edge protein‐modality photocleavage strategies: PhoCl, which cleaves a specific peptide bond directly upon photo irradiation, and LAUNCHER, which regulates enzymatic proteolysis through LOV2 conformational switching. Both technologies can be readily fused to proteins of interest and integrated into biological systems, making them user‐friendly and powerful tools for irreversible optical control. Nevertheless, systems constructed exclusively from natural amino‐acid sequences still face inherent constraints in system design flexibility, photosensitivity, and spectral tuning—limitations that remain key targets for future development.

## 3 Photocleavable Small Molecules

**Figure 4:**
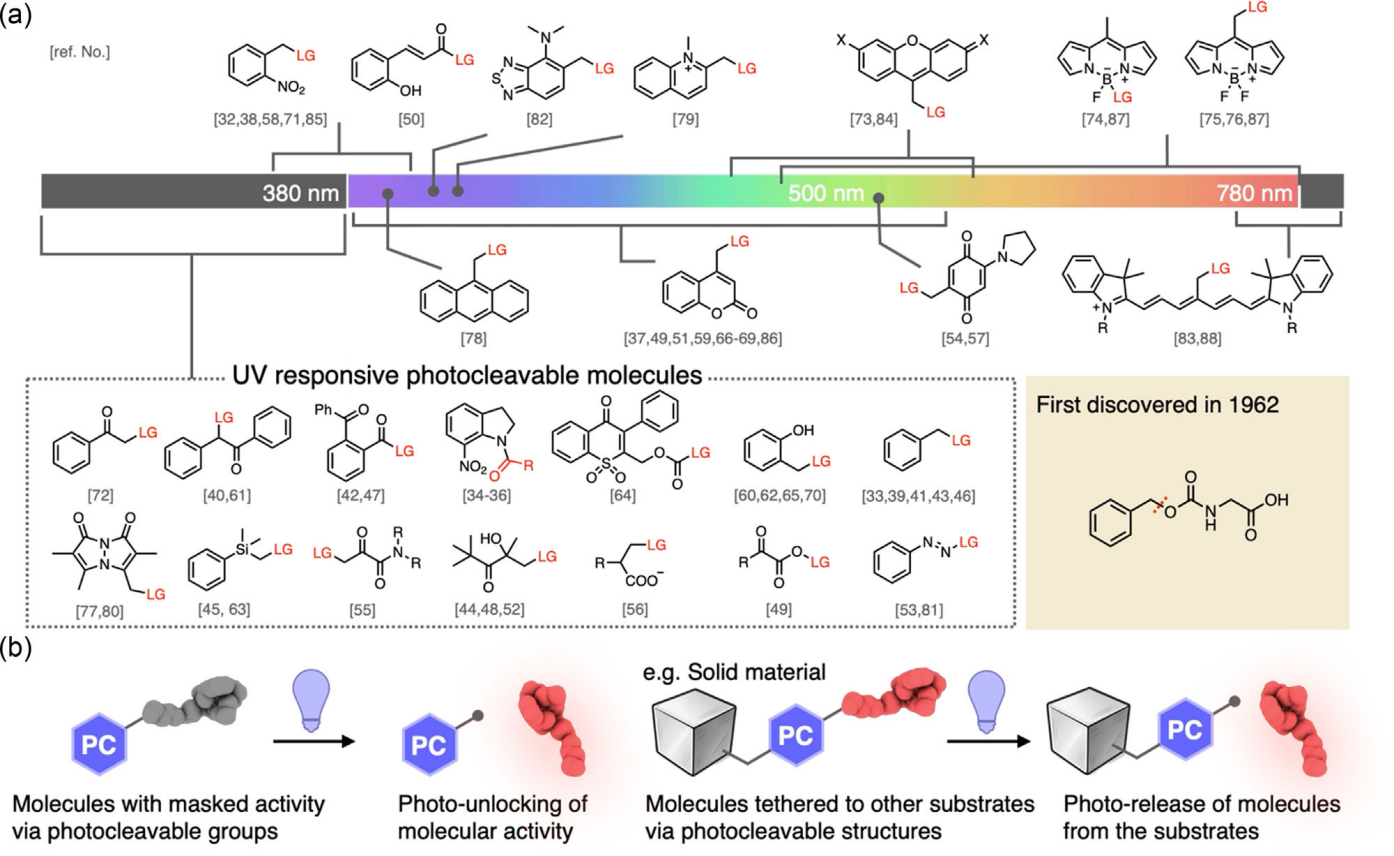
Small‐molecule photocleavable scaffolds and their applications. a) Representative photocleavable structures arrayed by absorption wavelength. b) Two canonical applications of photocleavable molecules. Photocaging: a photocage masks a molecule's activity until light exposure unmasks the protected functionality. Photo‐release: a photocage links a payload to a substrate (or another molecule) and, upon irradiation, releases the payload from the tether.

Beyond genetically encodable amino‐acid motifs, synthetically accessible small‐molecule photocages offer unrivaled structural diversity. Since benzyloxycarbonyl‐glycine was first identified as a photolabile protecting group in 1962,^[^ ^19^ ^]^ an ever‐expanding palette of core structures has emerged and found use from materials science to cell biology.^[^ ^12^, ^13^ ^–^ ^14^ ^,^ ^16^ ^,^ ^30^ ^,^ ^31^ ^]^ A compilation of the photocleavable core structures reported, along with their characteristic absorption wavelengths, is presented in **Figure** **4**a.^[^ ^13^ ^,^ ^14^ ^,^ ^32^, ^33^, ^34^, ^35^, ^36^, ^37^, ^38^, ^39^, ^40^, ^41^, ^42^, ^43^, ^44^, ^45^, ^46^, ^47^, ^48^, ^49^, ^50^, ^51^, ^52^, ^53^, ^54^, ^55^, ^56^, ^57^, ^58^, ^59^, ^60^, ^61^, ^62^, ^63^, ^64^, ^65^, ^66^, ^67^, ^68^, ^69^, ^70^, ^71^, ^72^, ^73^, ^74^, ^75^, ^76^, ^77^, ^78^, ^79^, ^80^, ^81^, ^82^, ^83^, ^84^, ^85^, ^86^, ^87^ ^–^ ^88^ ^]^ Driven by systematic structure–function exploration in organic synthesis, photocleavable motifs now span a broad spectral window; in recent years, scaffolds that undergo bond cleavage upon long‐wavelength near‐infrared (NIR) irradiation have also proliferated.^[^ ^83^ ^,^ ^89^, ^90^, ^91^ ^–^ ^92^ ^]^ For cellular applications, small‐molecule cages provide two decisive advantages: (i) tailor‐made structure‐function design enabled by modern synthetic chemistry, and (ii) wavelength agility achieved through modular substitution patterns, thereby enabling compatibility with multiphoton and near‐infrared activation, which is particularly useful for in vivo and deep‐tissue applications. We highlight the two canonical application strategies (Figure 4b): photo‐caging,^[^ ^93^, ^94^, ^95^, ^96^, ^97^ ^–^ ^98^ ^]^ in which a photocage masks molecular activity until light exposure unmasks the protected functionality, and photo‐release,^[^ ^81^ ^,^ ^99^, ^100^ ^–^ ^101^ ^]^ in which a photocage tethers a payload to a substrate and releases it on demand. In the subsections that follow, we focus on three mechanistically distinct scaffolds—nitrobenzyl, coumarin, and quinone—summarizing their key photochemical features and the specific advantages they bring to light‐controlled biological studies.

### 3.1 Nitrobenzene

**Figure 5:**
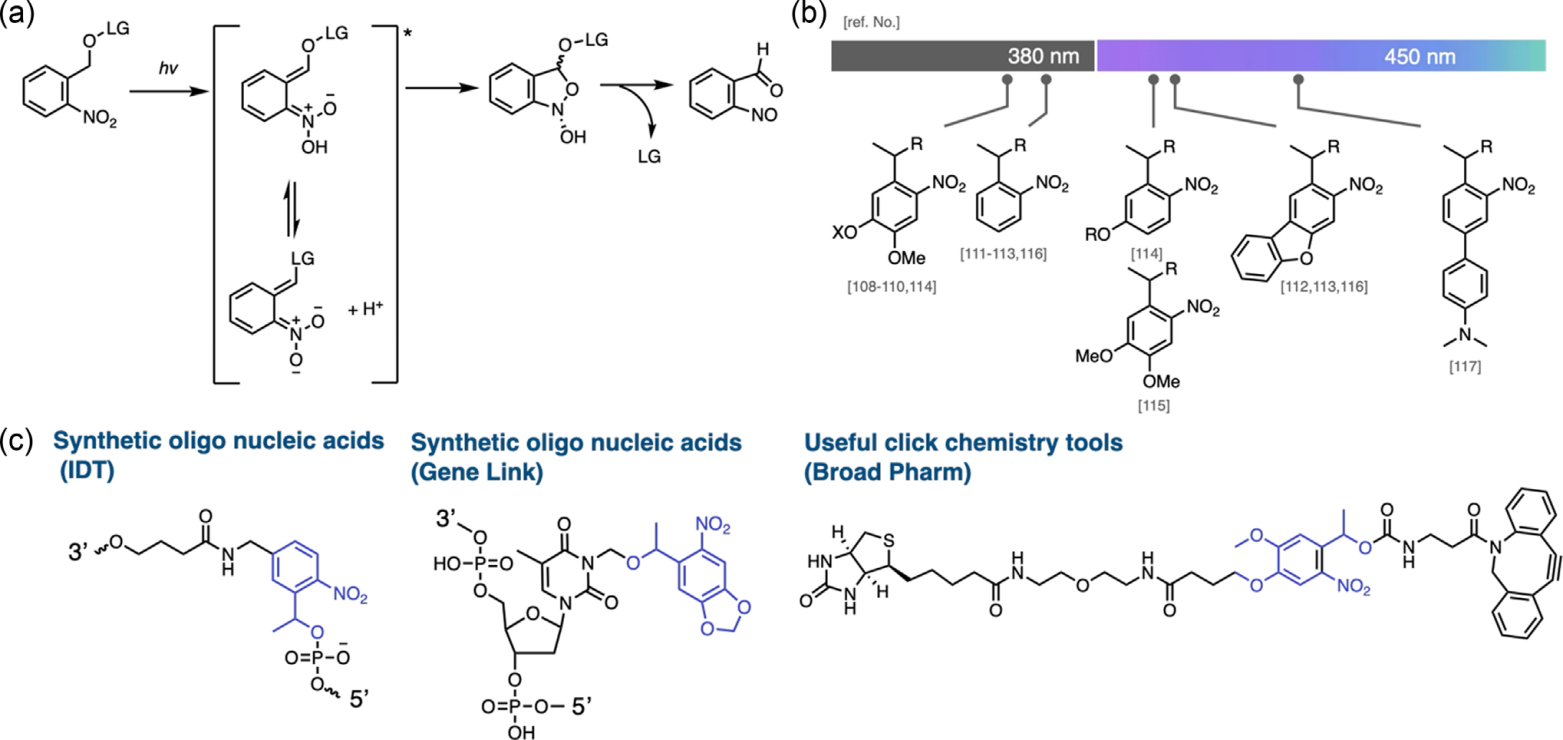
Overview of nitrobenzyl photocleavable systems. a) Proposed photocleavage mechanism of nitrobenzyl groups. b) Representative derivatives and their absorption wavelengths. c) Commercially available nitrobenzyl‐based photocaging tools for biological applications.

Nitrobenzyl groups are the most widely used class of photocleavable core structures. Since their first disclosure in 1965,^[^ ^32^ ^]^ they have been explored far beyond fundamental photochemistry, finding application in materials science and chemical biology.^[^ ^85^ ^,^ ^99^ ^,^ ^102^, ^103^ ^–^ ^104^ ^]^ Upon irradiation, a benzylic‐to‐nitro hydrogen transfer generates an aci‐nitro tautomer, which subsequently cyclises and undergoes heterolytic ring opening to release the leaving group (**Figure** **5**a).^[^ ^105^ ^]^ Due to their high photolytic efficiency and long‐standing track record, nitrobenzyl motifs are now offered as off‐the‐shelf options for custom oligonucleotide synthesis and are widely marketed as click chemistry reagents (Figure 5b).^[^ ^106^ ^,^ ^107^ ^]^ This ready commercial availability allows biologists and chemical biologists to integrate them into experiments with minimal synthetic effort. A key limitation, however, is their narrow spectral window: because the conjugated framework of nitrobenzyl groups is difficult to red‐shift, efficient cleavage still requires UV light (Figure 5c).^[^ ^108^, ^109^, ^110^, ^111^, ^112^, ^113^, ^114^, ^115^, ^116^ ^–^ ^117^ ^]^ In cellular contexts where visible‐light activation is preferred to minimize phototoxicity, this UV dependence remains a significant hurdle; to address this, recent strategies have employed two‐photon excitation systems or upconversion nanoparticles to enable longer‐wavelength activation and expand the biological applicability of nitrobenzyl groups.^[^ ^85^ ^]^

### 3.2 Coumarins

**Figure 6:**
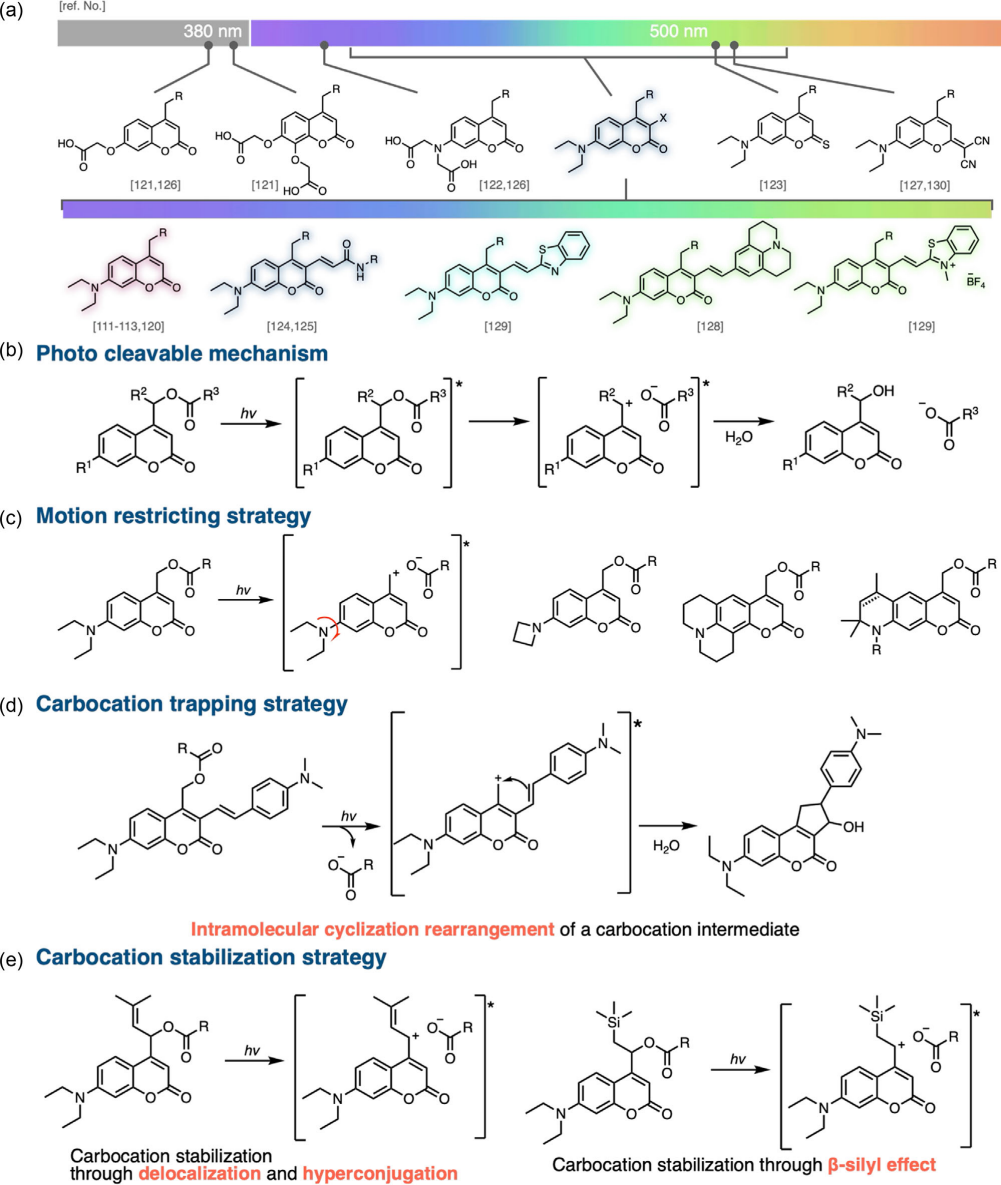
Overview of coumarin photocleavable systems. a) Representative derivatives and their absorption wavelengths. b) Proposed photocleavage mechanism of coumarins. c–e) Molecular design strategies that enhance photocleavage efficiency: c) rotational motion restriction, d) intramolecular carbocation trapping, and e) photogenerated carbocation stabilization.

Coumarin is the second most widely used photocleavable core structure after nitrobenzyl.^[^ ^86^ ^]^ Since its photolysis was first disclosed in 1984,^[^ ^37^ ^]^ research has progressed from fundamental structure–reactivity studies to broad applications as light‐responsive materials and prodrug‐type tools for cellular manipulation.^[^ ^118^ ^,^ ^119^ ^]^ Like nitrobenzyl photocages, coumarin core structures can be grafted onto both bioactive small molecules and biomacromolecules; importantly, their *π*‐conjugated scaffold is readily tunable, enabling systematic red‐shifts into the visible region.^[^ ^14^ ^]^ Numerous derivatives and their corresponding absorption and fluorescence have been reported (**Figure** **6**a).^[^ ^111^, ^112^ ^–^ ^113^ ^,^ ^116^ ^,^ ^120^, ^121^, ^122^, ^123^, ^124^, ^125^, ^126^, ^127^, ^128^, ^129^ ^–^ ^130^ ^]^ Unlike the photolytic pathway of nitrobenzyl groups, coumarin photolysis proceeds through an excited‐state cleavage that generates a contact ion pair; the generated carbocation is rapidly trapped by water (Figure 6b).^[^ ^131^, ^132^, ^133^, ^134^ ^–^ ^135^ ^]^ Leveraging this mechanism, several groups have recently engineered coumarin derivatives with higher photolytic efficiency. For example, transient‐absorption experiments on diethylaminocoumarins revealed that the diethylamino substituent undergoes excited‐state rotation, leading to the formation of a twisted intramolecular charge‐transfer (TICT) state. Restricting this rotation in tailored analogs effectively increased their fluorescence quantum yield and, consequently, enhanced their photocleavability (Figure 6c).^[^ ^136^, ^137^ ^–^ ^138^ ^]^ Other designs accelerate cleavage by trapping the photogenerated carbocation in a rapid intramolecular cyclization (Figure 6d).^[^ ^128^ ^]^ Most recently, two strategies have emerged to stabilize the photogenerated carbocation and thereby enhances photolysis efficiency. Feringa and coworkers exploited carbocation delocalization and hyperconjugation via an isobutenyl group,^[^ ^139^ ^,^ ^140^ ^]^ while Yoshimura and colleagues harnessed a *β*‐silyl effect introduced by a trimethylsilyl group.^[^ ^101^ ^]^ Earlier reports had already recognized the carbocation stabilization for improved photocleavage,^[^ ^141^ ^]^ but its significance and any further development remained largely overlooked until these recent breakthroughs.

### 3.3 Quinones

**Figure 7:**
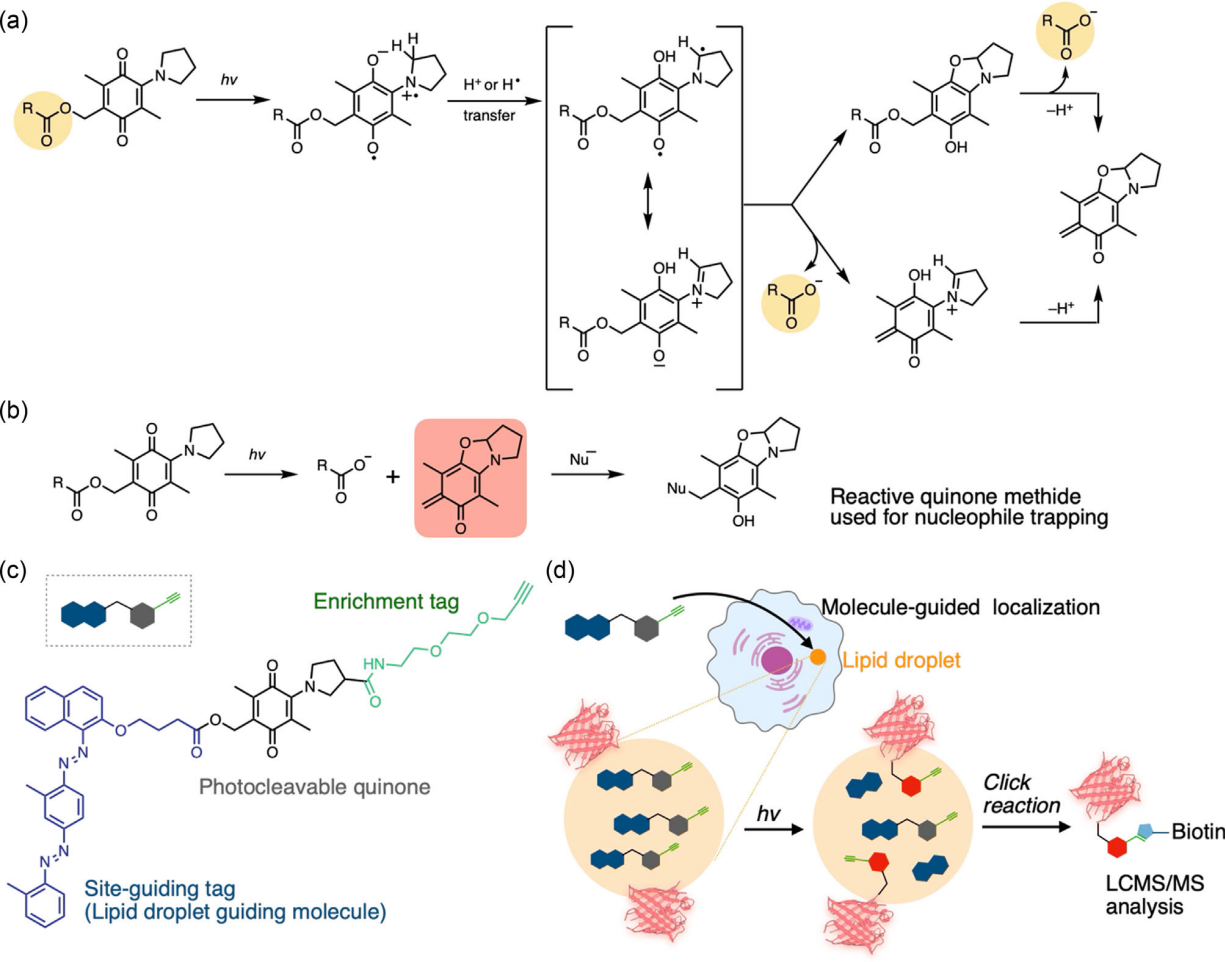
Example of quinone‐based photocleavable systems. a) Proposed photocleavage mechanism of quinone. b) In situ capture of a photo‐released *o*‐QM electrophile labels proximal nucleophile. c) A lipid‐droplet‐targeting aminobenzoquinone probe. d) Workflow for lipid‐droplet–targeted photocovalent labeling.

Quinone‐based photocages were first disclosed in 2005–2006 by Chen and Steinmetz, who demonstrated that 2‐pyrrolidino‐substituted 1,4‐benzoquinones undergo visible‐light activation to release carboxylate or phenolate leaving groups and generate an *o*‐quinone methide (*o*‐QM) electrophile.^[^ ^54^ ^,^ ^57^ ^]^ Detailed mechanistic studies have established a three‐step cascade (**Figure** **7**a): Norrish type II 1,5‐hydrogen shift: photo‐excitation (*λ* ≈ 450–550 nm) induces an intramolecular 1,5‐H shift, giving a biradical. Ion‐pair formation and cyclization: rapid electron transfer yields a zwitterionic ion pair that form a benzoxazoline through phenol‐assisted intramolecular cyclization. Payload release and *o*‐QM generation: Elimination from the benzoxazoline releases the leaving group and furnishes a highly reactive *o*‐QM. Quinone cages extend the traditional “photorelease” concept: the photoproduct itself can be exploited as a covalent probe (Figure 7b). A recent example involves a hydrophobic aminobenzoquinone probe that accumulates in lipid droplets and, upon light activation, releases an *o*‐QM that covalently tags nearby proteins, thereby enabling sub‐organelle mapping of the lipid‐droplet interactome in steatotic human liver tissue (Figure 7c,d).^[^ ^100^ ^]^ These studies illustrate how quinone photocages transcend simple payload release by harnessing the intrinsic reactivity of the cleavage byproduct, offering a versatile platform for proximity labeling and other chemoproteomic applications.^[^ ^100^ ^,^ ^142^ ^]^

In this section, we surveyed three classes of small‐molecule photocages that operate via distinct reaction mechanisms and highlighted their functional attributes. Small‐molecule platforms can be tuned to any excitation wavelength by selecting an appropriate core structure and auxiliary substituents, and they can be grafted onto diverse molecular frameworks for various applications. Furthermore, density functional theory (DFT) calculations can predict the detailed mechanisms of photolysis and directly inform structure–function–oriented molecular design.^[^ ^139^ ^,^ ^143^ ^,^ ^144^ ^]^ The most widely used system—nitrobenzyl derivatives—already benefits from robust commercial support. Modified DNA oligonucleotides and click chemistry tools bearing nitrobenzyl groups can be ordered off the shelf, minimizing the usage barrier for biologists. Coumarin‐based photocages are particularly attractive for molecular and cellular studies because they undergo efficient photolysis across the visible spectrum, thereby avoiding the phototoxicity associated with UV irradiation. Nevertheless, in most commonly employed core structures—including nitrobenzyl, coumarin, quinone, and related motifs—the cleavage site lies adjacent to a carbonyl group. Within biological media, such carbonyl linkages are susceptible to nonspecific hydrolysis, whereas structural modifications that improve hydrolytic robustness frequently attenuate the photolysis efficiency. Although this stability–reactivity tradeoff has been noted in the photomaterials literature, the issue has been largely overlooked in molecular and cell biology, and explicit design guidelines remain limited. Yoshimura and coworkers have addressed this gap by proposing design principles for biological applications and by introducing a remote silyl group that enhances hydrolytic stability and photoreactivity simultaneously.^[^ ^101^ ^]^ The continued refinement and adoption of such guidelines should foster the development of more powerful photocaging tools and, in turn, accelerate advances in molecular and cellular biology.

## 4 Photocleavable Metal Complexes

**Figure 8:**
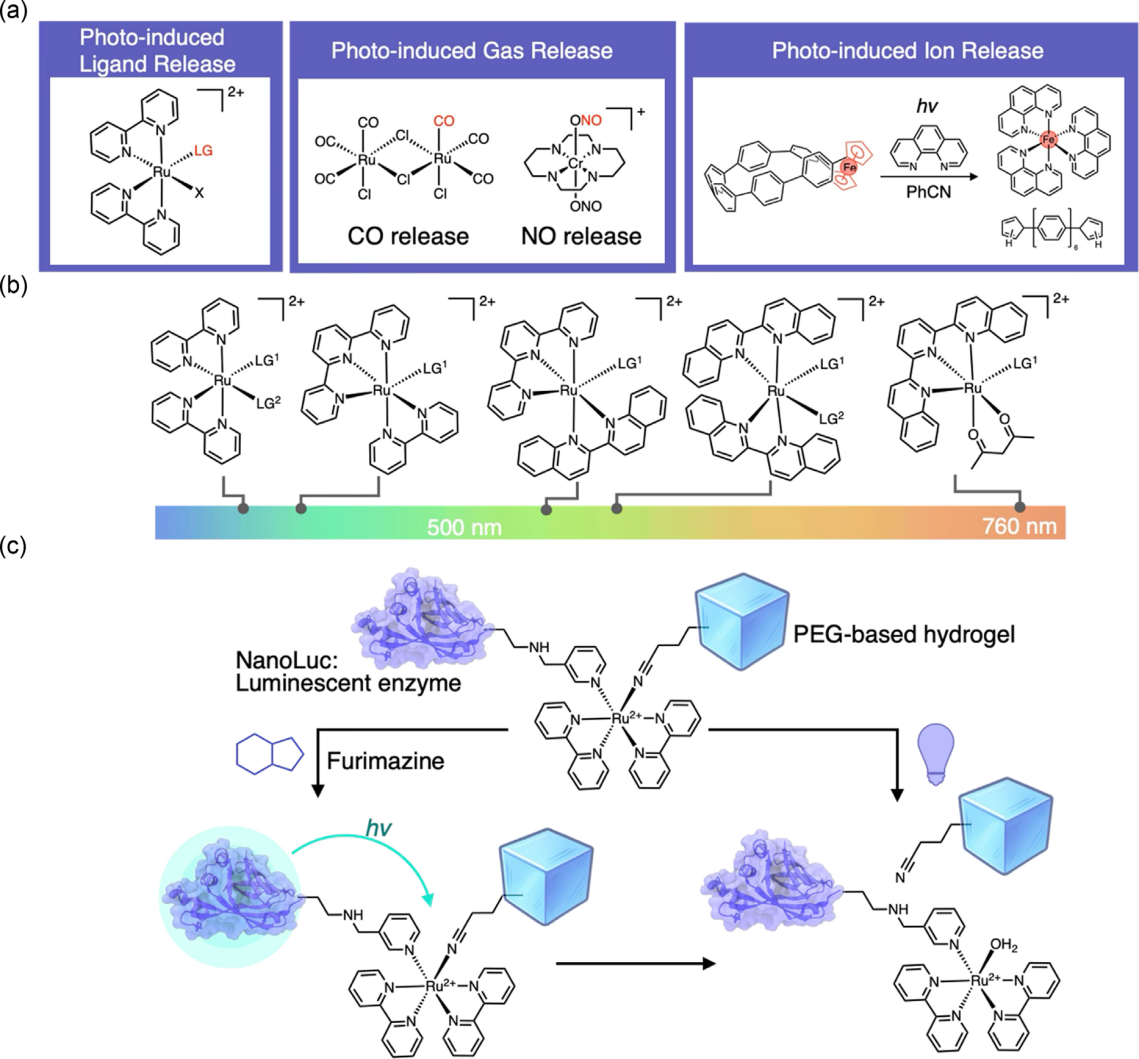
Example of photocleavable systems based on metal complexes. a) Representative examples of photo‐induced ligand‐, gas‐, and ion‐releasing complexes. b) Representative derivatives and their absorption wavelengths. c) Photo‐triggered protein‐releasing application using a photo‐cleavable metal complex.

As a newly emerging modality in photocleavable molecules, metal complexes have recently gained attention for their potential in chemical biology applications.^[^ ^145^ ^]^ Photocleavable metal complexes function via the photoinduced dissociation of ligands coordinated to the central metal atom (**Figure** **8**a). Early examples of such photoresponsive systems include potassium ferrioxalate (1953)^[^ ^146^ ^]^ and various oxalate complexes of Mn, Co, and Cr reported in the 1960s,^[^ ^147^ ^]^ which were primarily studied for their fundamental photochemical properties. The first biologically oriented photocleavable metal complexes, such as calcium photocages (1988)^[^ ^148^ ^]^ and the Pt(IV) complex [PtCl_2_I_2_(en)] (1996),^[^ ^149^ ^]^ enabled the release of Ca^2+^ ions and cisplatin analogs, respectively. However, the scope of these early systems was limited to specific ions or cytotoxic agents, restricting their broader applicability in chemical biology. In recent years, photolabile metal complexes have emerged as robust alternatives to traditional small‐molecule photocages, offering high aqueous stability and selective ligand dissociation under light. Their resistance to ligand exchange due to tightly bound ligands, and the absence of enzymatically labile carbonyl groups would provide key advantages for the design of photocaged compounds with enhanced stability in intracellular environments.

### 4.1 Ruthenium Complexes

Ruthenium(II) polypyridyl complexes are among the most widely used photocleavable metal complexes due to their favorable photophysical properties and compatibility with biological environments. Upon visible‐light excitation, these complexes undergo ligand dissociation through a well‐characterized metal‐to‐ligand charge transfer (MLCT) to a low‐lying triplet field (^3^LF) state transition.^[^ ^150^ ^]^ Originally explored for photocytotoxic applications via DNA binding,^[^ ^151^ ^]^ Ru(II) complexes have since been applied as photocages for optical controlling neuronal activity of *γ*‐aminobutyric acid (GABA)^[^ ^152^ ^]^ and dopamine.^[^ ^153^ ^]^ More recently, a significant advancement has been made in expanding this approach to the release of proteins. In 2024, Rapp and colleagues reported the development of a self‐illuminating photocaging system in which a ruthenium complex is photoactivated via bioluminescence resonance energy transfer (BRET) from a tethered luciferase (NanoLuc) (Figure 8c).^[^ ^154^ ^]^ In this system, light generated internally via substrate oxidation by NanoLuc efficiently triggers the dissociation of a Ru–ligand bond, resulting in the controlled release of a protein payload. Ru(II) complexes also offer the advantage of tunable absorption profiles via appropriate ligand design (Figure 8b).^[^ ^155^ ^,^ ^156^ ^]^ With the continued development of systems that respond efficiently to longer wavelengths, such as NIR light—which penetrates biological tissues more effectively—Ru‐based photocaged compounds are expected to find increasing utility in chemical biology and biomedical applications.^[^ ^157^ ^]^

### 4.2 Metal Complexes that Cage Nitric Oxide and Carbon Monoxide

Nitric oxide (NO) and carbon monoxide (CO) are gaseous signaling molecules (gasotransmitters) that exist only in trace amounts in cells, yet play vital roles in a range of physiological processes, including blood pressure regulation, neurotransmission, immune response, and inflammation suppression.^[^ ^158^ ^,^ ^159^ ^]^ To elucidate the detailed intracellular behavior of these gases, photoresponsive molecular tools capable of releasing these gaseous signaling molecules upon light irradiation are highly valuable. Both small organic molecules and metal complexes have been developed for this purpose.^[^ ^160^, ^161^, ^162^, ^163^, ^164^, ^165^ ^–^ ^166^ ^]^ Among these, metal complexes are particularly attractive because NO or CO can be directly coordinated to a metal center and selectively released upon light irradiation, allowing precise spatiotemporal control. Moreover, such complexes are often accessible without the need for advanced synthetic expertise and sophisticated molecular design, further enhancing their practical utility. For these reasons, here we focus on photolabile metal complexes as molecular tools for the light‐controlled release of NO and CO, which have been widely explored for dissecting their biological functions.^[^ ^161^ ^,^ ^163^ ^]^ Both NO and CO form stable M–NO and M–CO coordination bonds with metal centers, making metal complexes attractive structural platforms for the photorelease of these gas molecules (Figure 8a). Compared to organic photocaged compounds for NO and CO release, metal‐based complexes offer relatively straightforward synthesis and allow for the incorporation of multiple gas molecules per complex. NO‐releasing metal complexes (photo‐NORMs), including those based on Ru(II), Mn(I), and Cr(III), enable precise light‐controlled NO release.^[^ ^163^ ^,^ ^167^ ^]^ Notably, CrONO (trans‐[Cr(cyclam)(ONO)]^2+^) complexes offer high photoreactivity and thermal stability under physiological conditions.^[^ ^168^ ^,^ ^169^ ^]^ For CO release, Schatzschneider and colleagues introduced the first biocompatible photolabile CO‐releasing metal complex (photo‐CORM) based on the [Mn(CO)_3_]^+^ core.^[^ ^170^ ^]^ Recently, continued efforts have led to structural modifications such as Re–Mn dinuclear photo‐CORMs,^[^ ^171^ ^]^ and integration with upconversion nanoparticles^[^ ^172^ ^]^ to achieve near‐infrared (NIR) activation. These metal‐based gas donors provide controlled gas release and serve as powerful molecular tools to unravel gaseous signaling pathways in biology.

This section highlighted the emergence of metal complexes as a distinct class of photocleavable molecules with promising applications in chemical biology. Ruthenium‐based systems have evolved from DNA‐targeted phototherapeutics to platforms for controlled release of small neurotransmitters and even proteins. Likewise, photoactivatable metal–NO and metal–CO complexes enable precise delivery of gasotransmitters, with ongoing innovations in NIR‐responsive designs expanding their potential for deep‐tissue biological applications. In parallel, novel strategies have also demonstrated the feasibility of photocleavable systems for controlled metal‐ion release, exemplified by the recent green‐light‐induced Fe^2+^ uncaging from a ferrocene–cycloparaphenylene nanohoop (Figure 8a).^[^ ^173^ ^]^ These advances underscore the growing utility of metal‐based phototriggers for targeted, spatiotemporally controlled molecular interventions in living systems.

## 5 Summary and Outlook

**Figure 9:**
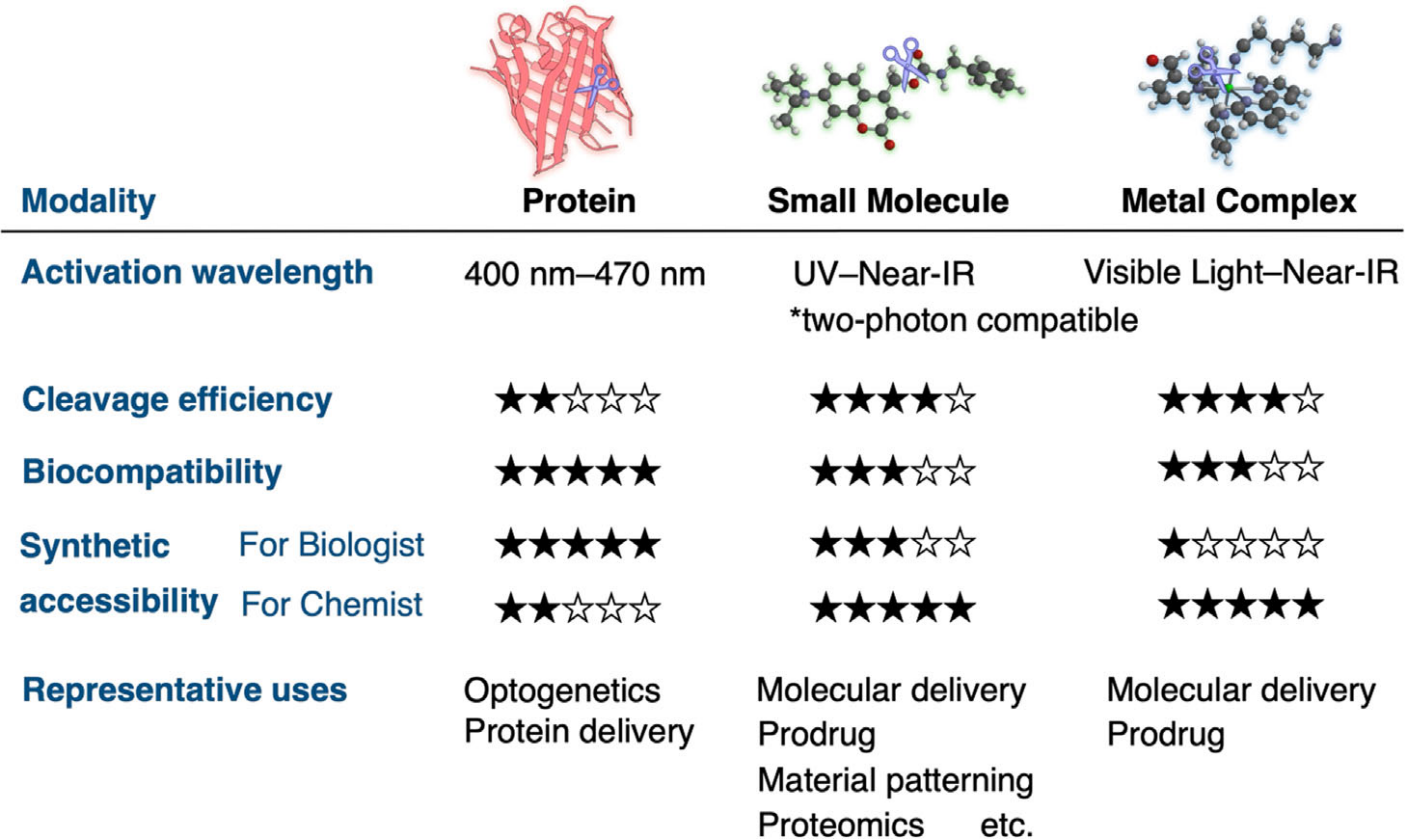
Comparative overview of photocleavable proteins, small molecules, and metal complexes based on key design features such as activation wavelength, cleavage efficiency, biocompatibility, synthetic accessibility, and representative uses.

Photocleavable tools for cellular control can be classified into three modalities—proteins, small molecules, and metal complexes—each evaluated through their wavelength tunability, cleavage efficiency, and biological compatibility, and synthetic accessibility (**Figure** **9**). Protein photocages offer plug‐and‐play genetic addressability and excellent biocompatibility but remain limited to blue/green excitation and moderate photoreactivity. Small‐molecule photocages exploit the full synthetic palette—atom‐level modularity, facile bioconjugation, and their photophysical properties can be rationally tuned through DFT‐guided design. However, they must still reconcile aqueous stability with rapid, low‐toxicity photolysis before they become truly plug‐and‐play reagents for any life‐science laboratory. Metal complexes unlock red/NIR activation, while further optimization is needed to minimize cytotoxicity and streamline synthesis. Despite their considerable potential in biological systems, reported examples remain scarce, and their limited accessibility continues to hinder broader use by biologists. Together, these three complementary modalities define a diverse molecular toolbox. Future advances will likely arise from hybrid systems that integrate their individual strengths—genetic addressability, tunable photochemistry, and long‐wavelength activation—paving the way toward universally accessible photocleavable reagents for biological research.

## Conflict of Interest

The authors declare no conflict of interest.

## Data Availability

The research data are not shared.
